# Multiple neural bHLHs ensure the precision of a neuronal specification event in *Caenorhabditis elegans*

**DOI:** 10.1242/bio.058976

**Published:** 2021-12-23

**Authors:** Konstantina Filippopoulou, Carole Couillault, Vincent Bertrand

**Affiliations:** Aix Marseille Univ, CNRS, IBDM, Turing Center for Living Systems, Marseille 13009, France

**Keywords:** *C. elegans*, Neuron, Neural bHLHs, Robustness

## Abstract

Neural bHLH transcription factors play a key role in the early steps of neuronal specification in many animals. We have previously observed that the Achaete-Scute HLH-3, the Olig HLH-16 and their binding partner the E-protein HLH-2 activate the terminal differentiation program of a specific class of cholinergic neurons, AIY, in *Caenorhabditis elegans*. Here we identify a role for a fourth bHLH, the Neurogenin NGN-1, in this process, raising the question of why so many neural bHLHs are required for a single neuronal specification event. Using quantitative imaging we show that the combined action of different bHLHs is needed to activate the correct level of expression of the terminal selector transcription factors TTX-3 and CEH-10 that subsequently initiate and maintain the expression of a large battery of terminal differentiation genes. Surprisingly, the different bHLHs have an antagonistic effect on another target, the proapoptotic BH3-only factor EGL-1, normally not expressed in AIY and otherwise detrimental for its specification. We propose that the use of multiple neural bHLHs allows robust neuronal specification while, at the same time, preventing spurious activation of deleterious genes.

## Abstract (precis)

**Summary:** During neuronal specification, the combined action of several neural bHLHs ensures the robust activation of terminal selector transcription factor expression and prevents the activation of deleterious genes.

## INTRODUCTION

Animals display complex behaviors. This functional complexity is mirrored by the high diversity of neuronal cell types of their nervous system. During development, the correct number of neurons of each neuronal cell type has to be produced so that the nervous system can function properly. How such a level of precision is reached in a robust manner remains poorly understood. The type-specific identity of a neuron is defined by the particular set of terminal selector transcription factors that it expresses (Deneris and Hobert, 2014; Hobert, 2011; Hobert and Kratsios, 2019). Terminal selectors are expressed throughout the life of the neuron and directly regulate the expression of large batteries of type-specific effector genes responsible for the functions of the neuron. During development, terminal selectors have to be activated in very precise sets of postmitotic neurons, at the right time and correct level. However, how this is achieved in a highly accurate manner is poorly characterized.

In animals, a conserved group of transcription factors, the neural bHLH factors, play key roles in early steps of nervous system development (Baker and Brown, 2018; Bertrand et al., 2002; Hartenstein and Stollewerk, 2015). They are involved in the choice between a neural and epidermal fate (classic proneural function), as well as in the specification of distinct neuronal-type identities. Neural bHLH factors include not only classic proneural bHLH factors but also bHLH factors involved in the establishment of the type-specific identity of the neuron. Neural bHLH factors are grouped in different families based on their sequences: Achaete-Scute, Neurogenin, Olig, Atonal and NeuroD. They act as heterodimers with another group of bHLH factors, the E-proteins, which are broadly expressed. Heterodimers of neural bHLHs with E-proteins bind to a specific DNA sequence, the E-box, to activate expression of their target genes. In this study, we analyzed how neural bHLH factors contribute to the precision in the initiation of terminal selector expression.

**Fig. 1.**
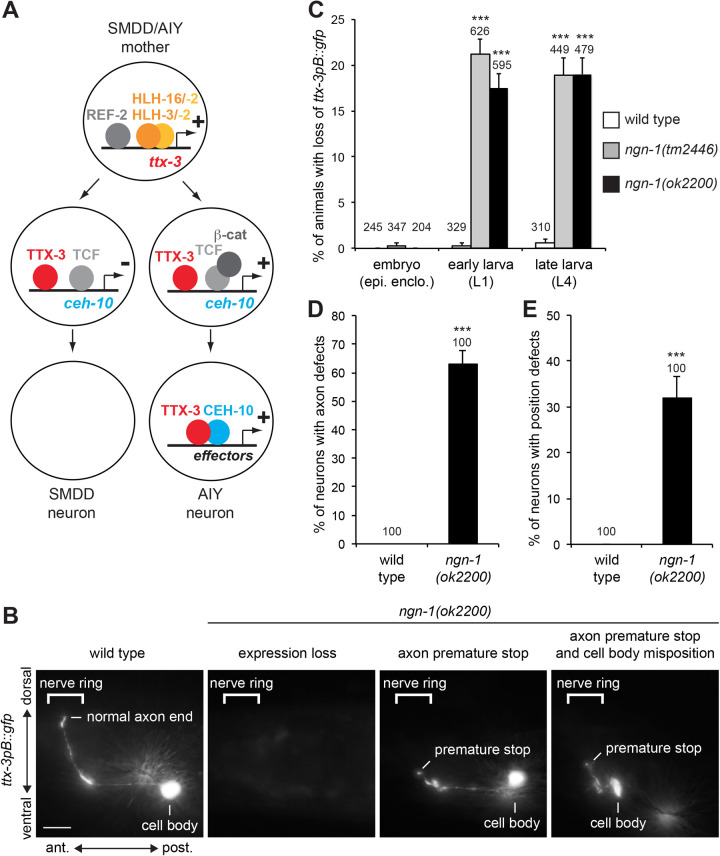
**Effect of *ngn-1* mutants on AIY neurons.** (A) Specification network of the AIY neuron. (B) Expression of *ttx-3pB::gfp (otIs173)* in AIY neurons of wild type or *ngn-1* mutant larvae (L4). Lateral view, anterior is left, dorsal is up, scale bar: 10 µm. (C) Percentage of animals with a loss of *ttx-3pB::gfp (otIs173)* expression in at least one AIY in wild type or *ngn-1* mutants, in embryos (epidermal enclosure), early larvae (L1) or late larvae (L4). (D) Percentage of AIY neurons expressing *ttx-3pB::gfp (otIs173)* with axonal projection defects in wild type or *ngn-1* mutants at late larval stage (L4). Axonal projection is considered defective if the axon stops before reaching the middle of the nerve ring. (E) Percentage of AIY neurons expressing *ttx-3pB::gfp (otIs173)* with cell body position defects in wild type or *ngn-1* mutants at late larval stage (L4). Position is considered defective if the cell body is located anterior to the posterior bulb of the pharynx. Error bars show standard error of proportion; numbers above the bars show numbers of animals or neurons analyzed; ***P<0.001, Fisher's exact test, a Bonferroni correction for multiple comparisons was applied in panel C.

To characterize the mechanisms that ensure the robustness of neuronal specification, we used *Caenorhabditis elegans* as a model organism. Each *C. elegans* adult hermaphrodite has exactly 302 neurons divided in 118 distinct classes (Hobert et al., 2016; White et al., 1986). It is therefore a good system to analyze how a nervous system is built with high precision. We used the AIY cholinergic interneuron as a test lineage, because its specification network is well characterized (Barriere and Bertrand, 2020). There are two AIY neurons, one on the left side of the head and one on the right. The specific identity of the AIY neuron is regulated by a complex of two terminal selector transcription factors, the homeodomain transcription factors TTX-3 (ortholog of LHX2/9) and CEH-10 (ortholog of VSX1/2) (Fig. 1A). In the postmitotic AIY neuron, TTX-3 and CEH-10 directly activate and maintain the expression of a large battery of type-specific effector genes such as the acetylcholine vesicular transporter *unc-17* or the neurotransmitter receptors *ser-2* and *mod-1* (Wenick and Hobert, 2004). In the embryo, the AIY neuron is produced during neurulation (epidermal enclosure) by an asymmetric division oriented along the anteroposterior axis (Sulston et al., 1983). This division generates the AIY neuron posteriorly and the SMDD motor neuron anteriorly. *ttx-3* expression is initiated in the SMDD/AIY mother cell by direct binding of several bHLH transcription factors to its *cis*-regulatory regions: the neural bHLH factors HLH-3 (Achaete-Scute family) and HLH-16 (Olig family), and their cofactor HLH-2 (E-protein) (Bertrand et al., 2011; Bertrand and Hobert, 2009; Murgan et al., 2015). The Zic transcription factor REF-2 also contributes to the initiation of *ttx-3* expression (Bertrand and Hobert, 2009). Following asymmetric division, the TTX-3 protein is inherited by both daughter cells. In the posterior daughter cell (AIY), the Wnt pathway is active leading to the formation of a TCF/β-catenin activator complex (POP-1/SYS-1) that acts with TTX-3 to directly activate *ceh-10* expression by binding to its *cis*-regulatory regions (Bertrand and Hobert, 2009). In the anterior daughter cell (SMDD), the Wnt pathway is inactive, β-catenin is degraded, and *ceh-10* expression is not activated. In the AIY neuron, following transcriptional activation, the CEH-10 protein forms a complex with TTX-3 that directly activates the expression of the AIY-specific effector gene battery (Wenick and Hobert, 2004). TTX-3 and CEH-10 also directly maintain their expression in the AIY neuron via a positive feedback loop throughout the life of the animal, locking in the type-specific identity of the neuron (Bertrand and Hobert, 2009; Wenick and Hobert, 2004).

In this study, we identify a third neural bHLH factor involved in AIY neuron specification, NGN-1, the only member of the Neurogenin family in *C. elegans*. By quantifying the initiation of the terminal selectors *ttx-3* and *ceh-10*, using CRISPR-engineered lines, we show that the three neural bHLH factors, NGN-1, HLH-3 and HLH-16 act together to set the correct level of terminal selector expression, ensuring robust, 100% efficient, neuronal specification. The neural bHLHs directly act on the terminal selector *cis*-regulatory regions via E-boxes. In addition, we show that HLH-3 and NGN-1 act in an antagonistic manner on the expression of another target, *egl-1*, a proapoptotic gene of the BH3-only family: HLH-3 promotes its expression while NGN-1 represses it. This antagonism blocks *egl-1* expression in the AIY neuron, preventing deleterious effects on AIY specification. Our study suggests that the use of several different neural bHLHs in a single neuron specification event allows the correct level of terminal selector expression to be reached, while simultaneously avoiding the ectopic activation of unwanted genes.

## RESULTS

### NGN-1 affects the development of the AIY neuron

In an RNAi screen for bHLH factors affecting the AIY neuron, we identified a role for NGN-1, the only Neurogenin ortholog in *C. elegans* (see Materials and Methods, Table S1). To confirm the implication of *ngn-1* we tested the effect of two deletion alleles, *tm2446*, a hypomorphic allele that deletes part of the bHLH domain, and *ok2200* a null allele that fully removes the bHLH domain (Nakano et al., 2010). We first analyzed the effect on the AIY neuron marker *ttx-3pB::gfp*, a transgene where *gfp* expression is driven by a *cis*-regulatory element of the terminal selector gene *ttx-3* (Altun-Gultekin et al., 2001; Bertrand and Hobert, 2009; Wenick and Hobert, 2004). At late larval stage (L4), we observed a partially penetrant loss of *ttx-3pB::gfp* expression in AIY with both alleles (Fig. 1B,C). The two alleles giving similar results, we decided to pursue the characterization with the *ok2200* null allele. When *ttx-3pB::gfp* is still expressed in AIY, we noticed that the axon is often shorter, stopping before reaching the dorsal side of the nerve ring (Fig. 1B,D). We also observed that the cell body is sometimes mispositioned, usually in a more anterior position (Fig. 1B,E). These data suggest that *ngn-1* affects the fate, axonal projection and position of the AIY neuron. Similar observations in *ngn-1* mutants have been recently reported (Christensen et al., 2020), however, in this study, the origin of these AIY neuron defects was not further analyzed.

To determine how *ngn-1* regulates AIY fate, we first analyzed the timing of *ttx-3pB::gfp* loss of expression (Fig. 1C). In wild-type animals, the expression of *ttx-3* is initiated in the embryo during neurulation (epidermal enclosure) and subsequently maintained in the early postmitotic AIY and throughout the life of the AIY neuron (Bertrand and Hobert, 2009). In *ngn-1* mutants, no loss of *ttx-3pB::gfp* is observed during neurulation in the AIY mother or early postmitotic AIY neuron. However, at hatching, in the early L1 larvae, *ttx-3pB::gfp* is lost in about 20% of animals, similar to what is observed in late larvae (L4). This suggests that the AIY neuron is normally generated during neurulation and loses *ttx-3pB::gfp* expression at later embryonic stages (before L1 larval stage). We then tested whether the loss of *ttx-3pB::gfp* expression could be a secondary consequence of a death of the AIY neuron rather than a loss of its identity. We labeled the AIY neurons with *ttx-3pB::gfp* and *ceh-10p::mcherry*, which allows to unambiguously follow the AIY neuron from its birth during neurulation until hatching. The AIY neuron is the only cell co-expressing both markers and, consistent with the cross-regulation between *ttx-3* and *ceh-10* during the maintenance phase (Bertrand and Hobert, 2009; Wenick and Hobert, 2004), when *ttx-3pB::gfp* expression is absent in *ngn-1* mutant larvae, *ceh-10p::mcherry* expression is also absent (Fig. S1). We did not observe any morphological sign of AIY cell death (neurite disappearance, cell rounding, refractile aspect under differential interference contrast optics) before the loss of AIY markers expression [embryos analyzed at fixed stages: 200 animals analyzed at stages between end of epidermal enclosure and 2-fold; 236 animals analyzed at stages between 2-fold and hatching; genotype: *ngn-1(ok2200); mgIs18(ttx-3pB::gfp); hpIs292(ceh-10p::mcherry)*]. This suggests that the loss of AIY markers is not a simple secondary consequence of a death of the neuron but rather reflects a defect in the AIY cell fate program.

### NGN-1 is transiently expressed in the AIY lineage during embryogenesis

**Fig. 2.**
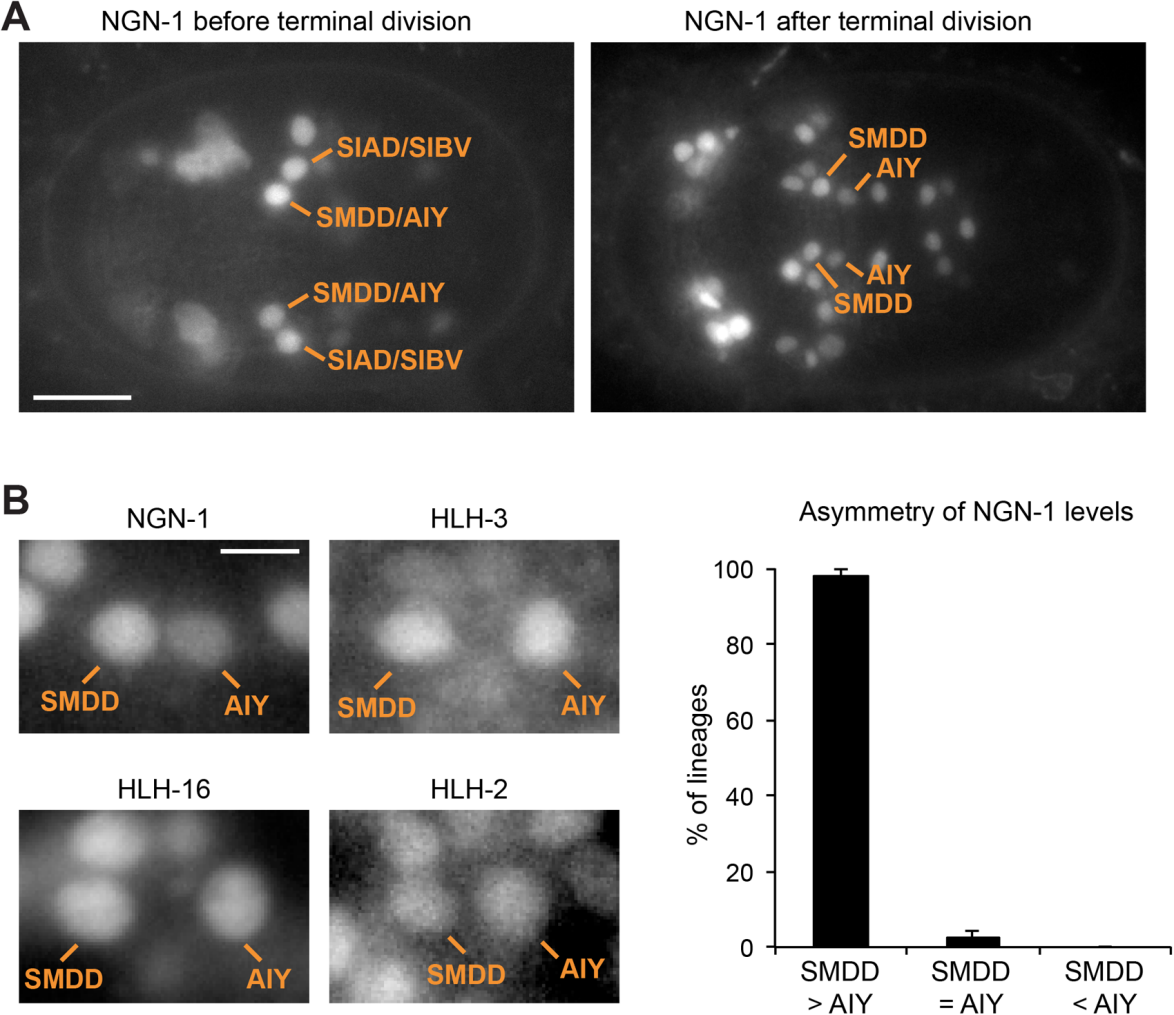
**Expression of bHLH factors in the AIY lineage.** (A) Expression of the NGN-1 protein tagged with GFP (*nIs394*) in embryos at epidermal enclosure stage before or after the terminal division of the SMDD/AIY mother cell. The AIY lineage is identified with *hlh-16p::mcherry (stIs10546)*. Ventral view, anterior is left, scale bar: 10 µm. (B) Pictures: expression in early postmitotic SMDD and AIY neurons of the NGN-1 protein tagged with GFP (*nIs394*), HLH-3 protein tagged with mNeonGreen (*vlc28*), HLH-16 protein tagged with GFP (*otEx4503*), and HLH-2 protein tagged with GFP (*nIs407*). The SMDD and AIY neurons are identified with *ttx-3pB::mcherry (otIs181* or *otEx2644)*. Epidermal enclosure stage, ventral view, anterior is left, scale bar: 2 µm. Graph: percentage of lineages with a higher level of NGN-1 protein in SMDD than AIY (SMDD>AIY), similar level (SMDD=AIY) or lower level (SMDD<AIY). Error bars show standard error of proportion; *n*=44 lineages analyzed.

To further characterize how NGN-1 regulates AIY cell fate, we analyzed its expression in the AIY lineage. We used a rescuing translational protein fusion, NGN-1::GFP (*nIs394*) (Nakano et al., 2010). We observed that NGN-1 is expressed during embryogenesis in the SMDD/AIY mother cell before its terminal division as well as in its sister cell, the SIAD/SIBV mother cell (Fig. 2A, cells identified by co-labelling with *hlh-16p::mcherry*, *stIs10546*). Following the terminal division, NGN-1 is detected in both the early postmitotic AIY neuron and early postmitotic SMDD neuron (Fig. 2A). NGN-1 subsequently disappears from the AIY neuron, being undetectable in AIY at larval stages. This transient expression in the AIY lineage is consistent with a role of NGN-1 in the regulation of AIY fate during embryogenesis, before L1 larval stage. We did not observe any difference in NGN-1 expression between the left and right AIY lineages (Fig. S2A). In addition, *ngn-1* mutants affect the left and right AIY neurons in a similar manner (Fig. S2B).

We have previously observed that three other bHLH factors (HLH-3/Achaete-Scute, HLH-16/Olig and HLH-2/E) are expressed in the SMDD/AIY mother cell during embryogenesis, where they contribute to the initiation of *ttx-3* expression (Murgan et al., 2015). Interestingly, following division, we observed that HLH-3 is enriched in the anterior daughter cell (SMDD/AIY mother cell) when compared to the posterior daughter cell (SIAD/SIBV mother cell) in a Wnt pathway regulated manner (Murgan et al., 2015). On the contrary, HLH-16 and HLH-2 have similar levels between the SMDD/AIY mother and SIAD/SIBV mother (Murgan et al., 2015). We therefore looked at whether NGN-1 could display asymmetric levels between the SMDD/AIY mother and SIAD/SIBV mother, but we did not observe any consistent difference. However, when analyzing the subsequent division (terminal division of the SMDD/AIY mother cell), we observed that NGN-1 is enriched in the anterior daughter cell (SMDD neuron) when compared to the posterior daughter cell (AIY neuron) (Fig. 2B). We then tested whether the three other bHLH factors could also be asymmetric during this terminal division by looking at the endogenous HLH-3 protein tagged with mNeonGreen (*vlc28*; Lloret-Fernández et al., 2018) or rescuing translational GFP protein fusions of HLH-16 (*otEx4503*; Bertrand et al., 2011) and HLH-2 (*nIs407*; Nakano et al., 2010). However, none of them displayed asymmetries between the early postmitotic SMDD and AIY neurons (Fig. 2B). Taken together, these data suggest that bHLH factors are differentially regulated in Wnt controlled asymmetric divisions: HLH-3 is asymmetric in the SMDD/AIY versus SIAD/SIBV division, while NGN-1 is asymmetric in the subsequent SMDD versus AIY division.

### The bHLH factors NGN-1, HLH-3 and HLH-16 act together to specify the AIY neuron

**Fig. 3.**
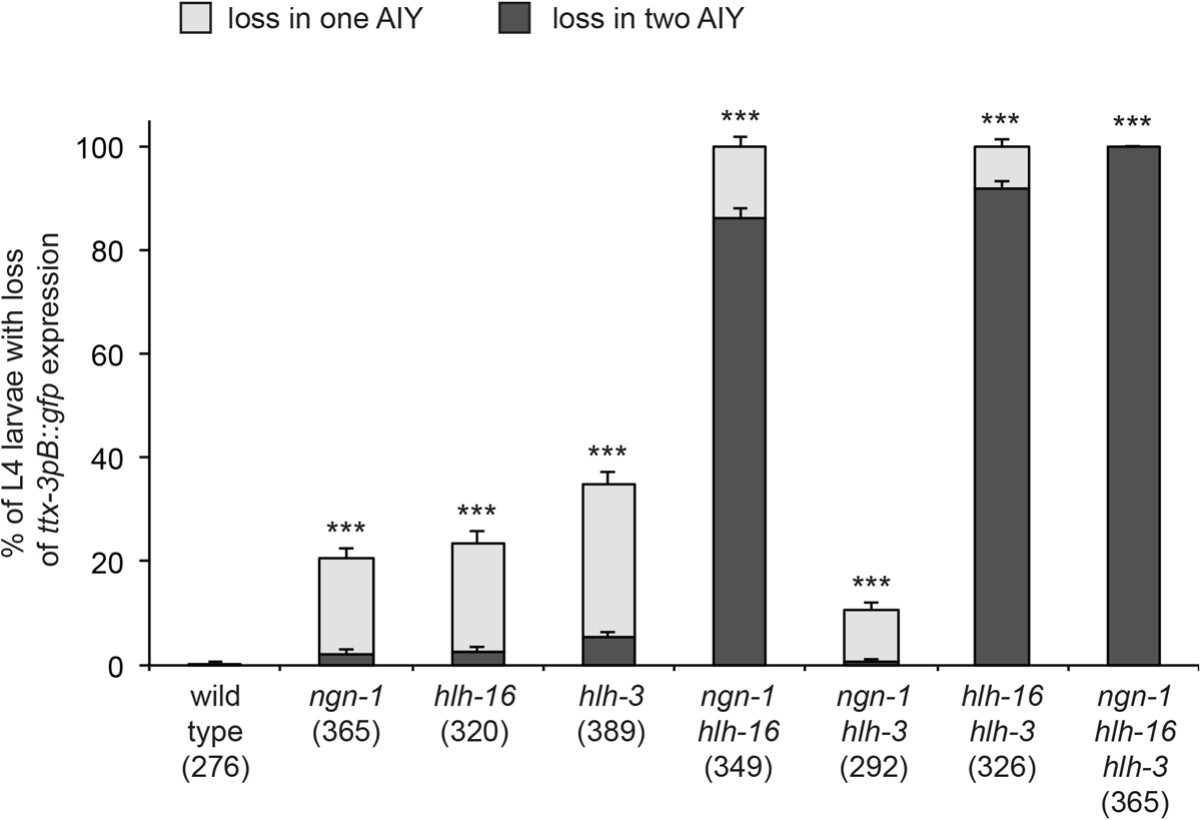
**Effect of combinations of bHLH mutants on AIY neurons at larval stage.** Percentage of animals with a loss of *ttx-3pB::gfp (otIs173)* expression in one or both AIY neurons at late larval stage (L4) in wild type or combinations of *ngn-1(ok2200)*, *hlh-16(ot711)* and *hlh-3(tm1688)* mutants. Error bars show standard error of proportion; numbers below genotypes show numbers of animals analyzed. Statistics: each mutant genotype is different from wild type (***P<0.001), in addition, each double mutant is different from the corresponding single mutants (*P*<0.01, enhancement for *ngn-1; hlh-16* and *hlh-16; hlh-3*, suppression for *ngn-1; hlh-3*), the triple mutant is different from the double mutants (*P*<0.001, enhancement), Fisher's exact test with Bonferroni correction for multiple comparisons.

We have previously observed that, in loss-of-function mutants of *hlh-3* or *hlh-16*, there is a partially penetrant loss of *ttx-3* expression in the AIY neuron (Murgan et al., 2015), similar to what we observed here in *ngn-1* mutants. To characterize the interaction between these three bHLH factors, we analyzed the effect on *ttx-3pB::GFP*, at L4 larval stage, of combinations of null mutants: *ngn-1(ok2200)*, *hlh-16(ot711)* and *hlh-3(tm1688)* (Fig. 3). While each single mutant generates a partially penetrant loss of *ttx-3pB::gfp* expression, only the triple mutant produces a fully penetrant loss. This suggests that the combined action of the three bHLH factors is required to specify the AIY neuron in a 100% efficient manner, and that they have a partially redundant function. Interestingly, while the penetrance is enhanced in the double *ngn-1; hlh-16* mutant or the double *hlh-16; hlh-3* mutant relative to single mutants, this is not the case in the double *ngn-1; hlh-3* mutant, suggesting that the interaction between *ngn-1* and *hlh-3* is more complicated than a simple redundancy. Neurogenin, Achaete-Scute and Olig family bHLH factors (like NGN-1, HLH-3 and HLH-16) usually act as heterodimers with E/Daughterless bHLH factors (Bertrand et al., 2002). *Caenorhabditis elegans* has only one E/Daughterless family member, HLH-2. We have previously observed that, when *hlh-2* is knocked down using RNAi, *ttx-3pB::GFP* expression is lost in a nearly fully penetrant manner (Bertrand and Hobert, 2009). This suggests that three bHLH complexes (NGN-1:HLH-2, HLH-3:HLH-2 and HLH-16:HLH-2) act together to specify the AIY neuron.

### The bHLH factors NGN-1, HLH-3 and HLH-16 differentially affect the initiation of the terminal selectors TTX-3 and CEH-10

To determine how NGN-1, HLH-3 and HLH-16 affect the specification of the AIY neuron, we then analyzed their effects on the initiation of expression of the terminal selector transcription factors TTX-3 and CEH-10 in the AIY lineage during embryogenesis. To quantify their effects, we used quantitative imaging of animals, where the endogenous TTX-3 or CEH-10 proteins have been tagged with YFP by CRISPR genome engineering of the endogenous locus: *ttx-3endo::yfp (vba3)* or *ceh-10endo::yfp (vba1)*. When analyzed during the maintenance phase at L4 larval stage, *ttx-3endo::yfp* (Fig. S3) gave similar results to *ttx-3pB::gfp* (Fig. 3).

**Fig. 4.**
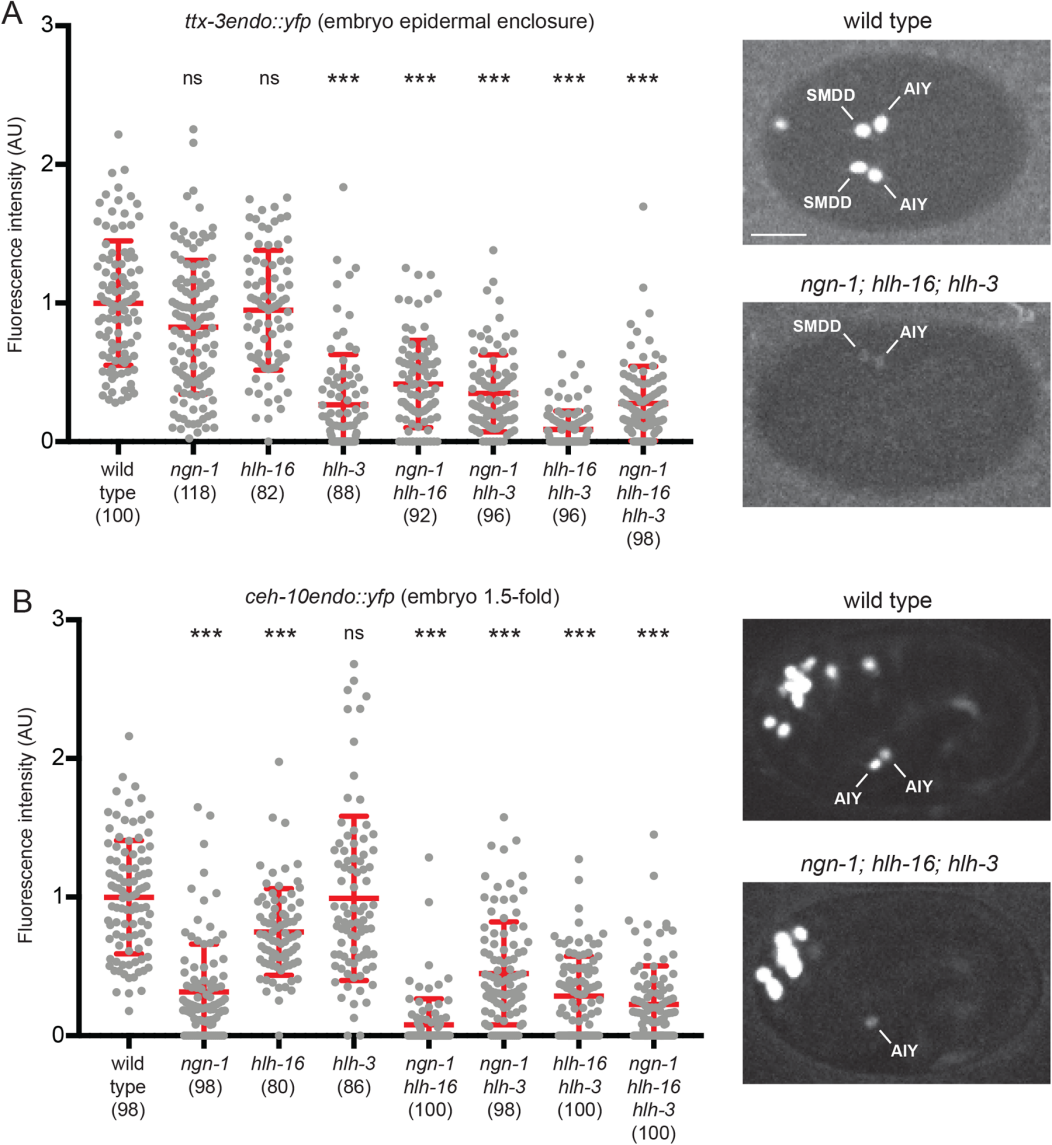
**Effect of combinations of bHLH mutants on the initiation of *ttx-3* and *ceh-10* expression in the embryo.** (A) Graph: quantification of the fluorescence levels of *ttx-3endo::yfp (vba3)* in AIY neurons at epidermal enclosure embryonic stage in wild type or combinations of bHLH mutants. Each grey dot represents one neuron. The number of neurons analyzed is presented below the genotype. Red bars represent mean and s.d., Mann–Whitney test with Bonferroni correction for multiple comparisons (ns: not significant, ***P<0.001). Please note that, in *ngn-1* single mutants, only a decrease of *ttx-3endo::yfp* expression intensity is observed (but no complete loss) at epidermal enclosure stage; these data are consistent with the observation of no complete loss of *ttx-3pB::gfp* expression at epidermal enclosure stage reported in Fig. 1C. Pictures: expression of *ttx-3endo::yfp (vba3)* at epidermal enclosure embryonic stage. The wild-type embryo shows strong expression in the two SMDD and AIY neurons. The *ngn-1; hlh-16; hlh-3* triple mutant embryo shows weak expression in one SMDD and AIY, and no expression in the other SMDD and AIY. Ventral view, anterior is left, scale bar: 10 µm. (B) Graph: quantification of the fluorescence levels of *ceh-10endo::yfp (vba1)* in AIY neurons at 1.5-fold embryonic stage in wild type or combinations of bHLH mutants. Pictures: expression of *ceh-10endo::yfp (vba1)* at 1.5-fold embryonic stage. The wild-type embryo shows strong expression in the two AIY neurons. The *ngn-1; hlh-16; hlh-3* triple mutant embryo shows weaker expression in one AIY, and no expression in the other. Lateral view, anterior is left, dorsal is up.

We first looked at the effect of bHLH mutants on the initiation of TTX-3 expression. TTX-3 expression starts in the AIY lineage during epidermal enclosure embryonic stage (neurulation). In the *hlh-3* mutant, we observed a strong reduction of TTX-3 expression level (Fig. 4A). While in *ngn-1* or *hlh-16* single mutants TTX-3 expression is not highly affected, we observed a strong reduction in the *ngn-1; hlh-16* double mutant, suggesting that NGN-1 and HLH-16 also positively regulate TTX-3 expression.

We then looked at the effect on the initiation of CEH-10 expression. CEH-10 expression starts in AIY a bit later than TTX-3, at the 1.5-fold embryonic stage. In the *ngn-1* mutant, we observed a strong reduction of CEH-10 expression level (Fig. 4B). We also observed a weaker reduction in the *hlh-16* mutant. Finally, while the *hlh-3* mutant alone shows no defect, we observed a strong reduction when *hlh-3* and *hlh-16* mutations are combined, suggesting that HLH-3 also positively regulates CEH-10 expression.

Taken together, these data suggest that NGN-1, HLH-3 and HLH-16 act together to set up the correct level of expression of the two terminal selectors TTX-3 and CEH-10 during the initiation phase in the embryo. HLH-3 seems to be the most important for TTX-3 initiation and NGN-1 the most important for CEH-10 initiation. We noticed, however, that in the triple *ngn-1; hlh-16; hlh-3* mutant some AIY neurons still show initiation of TTX-3 or CEH-10 expression, although the level is often reduced (Fig. 4A,B). In these AIY neurons that still initiate TTX-3 and CEH-10 expression, the levels may not be high enough to trigger the automaintenance loop, leading to a failure of AIY fate acquisition and absence of *ttx-3pB::gfp* or *ttx-3endo::yfp* expression at L4 larval stage (Fig. 3; Fig. S3).

**Fig. 5.**
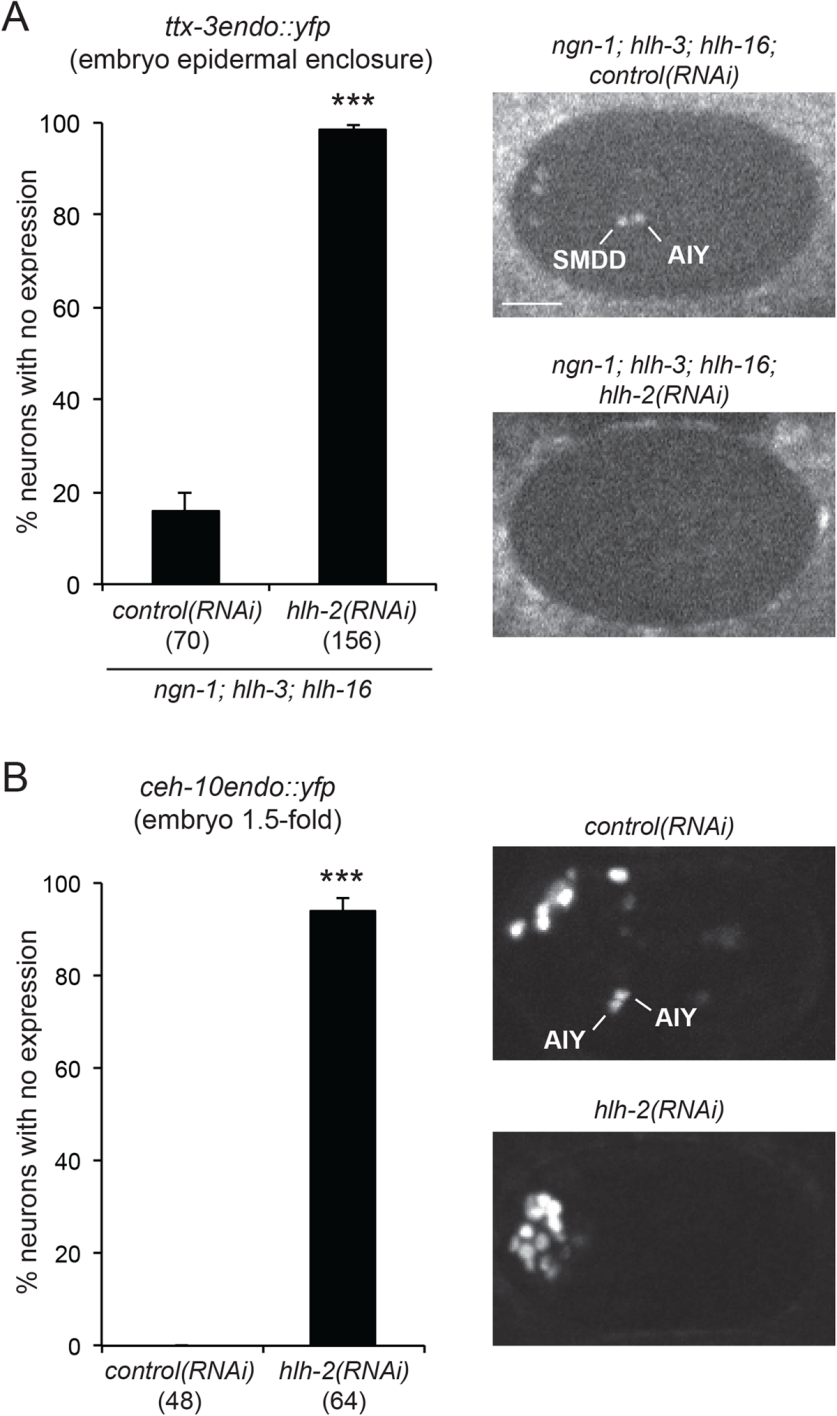
**Effect of *hlh-2* on the initiation of *ttx-3* and *ceh-10* expression in the embryo.** (A) Graph: percentage of AIY neurons with no expression of *ttx-3endo::yfp (vba3)* in *ngn-1(ok2200)*; *hlh-16(ot711)*; *hlh-3(tm1688)* triple mutants treated with *control(RNAi)* or *hlh-2(RNAi)*. Embryos at epidermal enclosure stage; error bars show standard error of proportion; numbers below genotypes show numbers of neurons analyzed; Fisher's exact test (***P<0.001). Pictures: expression of *ttx-3endo::yfp (vba3)* at epidermal enclosure embryonic stage. The *ngn-1; hlh-16; hlh-3* triple mutant embryo treated with *control(RNAi)* shows weak expression in one SMDD and AIY, and no expression in the other SMDD and AIY. The *ngn-1; hlh-16; hlh-3* triple mutant embryo treated with *hlh-2(RNAi)* shows no expression in SMDD or AIY. Ventral view, anterior is left, scale bar: 10 µm. (B) Graph: percentage of AIY neurons with no expression of *ceh-10endo::yfp (vba1)* after treatment with *control(RNAi)* or *hlh-2(RNAi)*. To increase RNAi efficiency, the animals contain the *rrf-3(pk1426)* mutation. Embryos at 1.5-fold stage; error bars show standard error of proportion; numbers below genotypes show numbers of neurons analyzed; Fisher's exact test (***P<0.001). Pictures: expression of *ceh-10endo::yfp (vba1)* at 1.5-fold embryonic stage. The embryo treated with *control(RNAi)* shows strong expression in the two AIY neurons. The embryo treated with *hlh-2(RNAi)* shows no expression in AIY. Lateral view, anterior is left, dorsal is up.

We have previously observed that RNAi knockdown of *hlh-2* leads to a loss of *ttx-3pB::gfp* initiation at epidermal enclosure embryonic stage (Bertrand and Hobert, 2009), showing that HLH-2 is required for the initiation of *ttx-3* expression in the embryo. HLH-2 usually acts as a heterodimer with class II (tissue specific) bHLH factors such as neural bHLH factors (Grove et al., 2009; Krause et al., 1997; Nakano et al., 2010), and therefore likely acts as heterodimer with HLH-3, NGN-1 and HLH-16 in wild-type conditions. However, it has been observed that, when class II bHLH factors are absent, HLH-2 is able to act as a homodimer (Sallee and Greenwald, 2015), although this is not its main mode of action. We were therefore wondering whether HLH-2 could be responsible for the remaining initiation level of TTX-3 and CEH-10 in the triple *ngn-1; hlh-16; hlh-3* mutant. To test this, we performed an RNAi knockdown of *hlh-2* in an *ngn-1; hlh-16; hlh-3* triple mutant background and tested the effect on the initiation of TTX-3 expression (Fig. 5A). While, in the *ngn-1; hlh-16; hlh-3* triple mutant, 80% of AIY neurons still display some level of TTX-3 initiation, *hlh-2* knockdown completely eliminates the remaining expression in nearly all AIY neurons, showing that it is dependent on HLH-2. We could not conduct the same experiment on CEH-10 initiation because 1.5-fold embryos of the *ngn-1; hlh-16; hlh-3* triple mutant treated with *hlh-2* RNAi are too disorganized to allow unambiguous identification of the AIY neuron. We therefore tested the effect of *hlh-2* RNAi in the absence of the *ngn-1; hlh-16; hlh-3* mutations (Fig. 5B). We observed that, in *hlh-2* RNAi animals, CEH-10 initiation is completely eliminated in nearly 100% of the AIY neurons, showing that HLH-2 is essential for CEH-10 activation.

### The *cis*-regulatory regions of *ttx-3* and *ceh-10* contain putative binding sites for NGN-1, HLH-3 and HLH-16

**Fig. 6.**
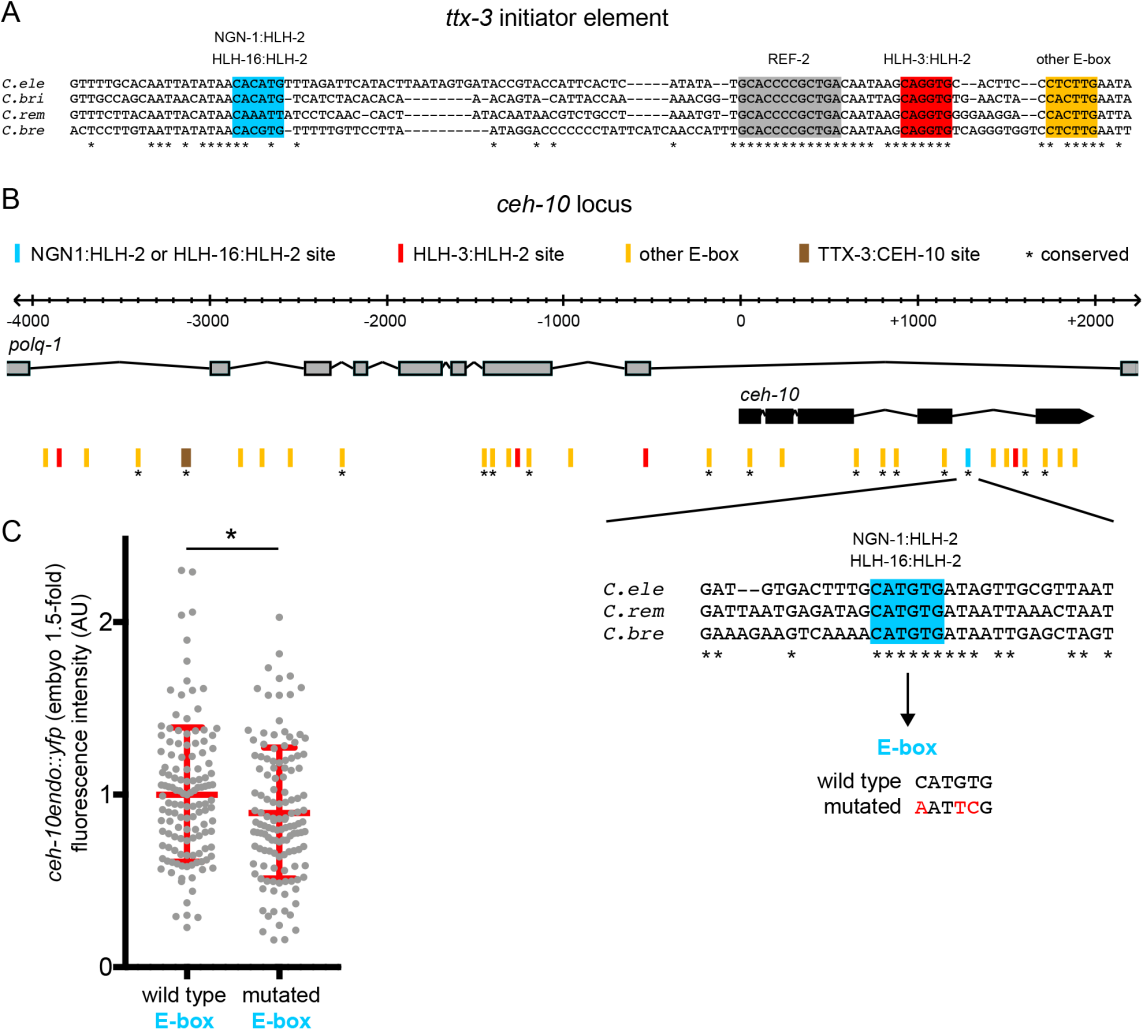
**bHLH binding sites in the *ttx-3* and *ceh-10 cis*-regulatory regions.** (A) Sequence of the *cis*-regulatory region that regulates the initiation of *ttx-3* expression in the AIY lineage. (B) Region of the *ceh-10* locus sufficient to rescue a *ceh-10* mutant. *ceh-10* is located in introns of another gene (*polq-1*) in the opposite direction. *: bHLH binding site conserved between *C. elegans* (*C. ele*) and at least another species among *C. briggsae* (*C. bri*), *C. remanei* (*C. rem*) and *C. brenneri* (*C. bre*). (C) Quantification of the fluorescence levels of *ceh-10endo::yfp (vba1)* wild type or mutated for the conserved CATGTG E-box (*vba28*). Each grey dot represents one neuron. Red bars represent mean and s.d., *n*=130 neurons analyzed for each genotype, Mann–Whitney test (*P<0.05).

We then analyzed whether NGN-1, HLH-3 and HLH-16 could directly activate *ttx-3* and *ceh-*10 expression. bHLH transcription factors of the Neurogenin, Achaete-Scute or Olig families (like NGN-1, HLH-3 and HLH-16) usually act as heterodimers with E-proteins (like HLH-2) by binding to E-box sequences (CANNTG) present in *cis*-regulatory regions (Bertrand et al., 2002). The two central nucleotides (NN) differ depending on the bHLH dimer considered, and the preference of various bHLH dimers of *C. elegans* for specific E-box sequences has been established (De Masi et al., 2011; Grove et al., 2009). We have previously identified a 113 bp *cis*-regulatory element responsible for the initiation of *ttx-3* expression in the AIY lineage at epidermal enclosure stage (*ttx-3* initiator element; Bertrand and Hobert, 2009). This region contains three E-boxes, partially conserved between different *Caenorhabditis* species, and mutations of these E-boxes suppress its activity (Bertrand and Hobert, 2009). Interestingly, according to *C. elegans* bHLH binding preference rules established in large scale studies (De Masi et al., 2011; Grove et al., 2009), the sequence of the best conserved E-box (CAGGTG) in the *ttx-3* initiator element corresponds to the preference of the HLH-3:HLH-2 complex, while the sequence of a less well conserved E-box (CACATG) corresponds to the preference of the NGN-1:HLH-2 and HLH-16:HLH-2 complexes (Fig. 6A). This suggests that NGN-1, HLH-3, HLH-16 and HLH-2 could possibly directly regulate the initiation of *ttx-3* expression.

We have previously observed that TTX-3 contributes to the initiation of CEH-10 expression (Bertrand and Hobert, 2009), therefore part of the effect observed on CEH-10 initiation in bHLH mutants could be indirect via TTX-3. However, while *ceh-10endo::yfp* expression is completely abolished in *ttx-3* mutants during the maintenance phase (L4 larval stage), expression is reduced but not completely lost during the initiation phase (1.5-fold embryo), suggesting that other factors are acting in parallel to TTX-3 to initiate *ceh-10* expression (Fig. S4). In addition, we noticed that *ngn-1* or *hlh-16* single mutants have no or only little effect on the initiation of TTX-3 expression, while they strongly affect the initiation of CEH-10 expression (Fig. 4). This suggests that NGN-1 and HLH-16 may act on CEH-10 expression in part independently of TTX-3. We therefore looked at whether they could directly regulate the initiation of CEH-10 expression. The *ceh-10* genomic region of 6 kb, sufficient to rescue AIY development in a *ceh-10* mutant (Bertrand and Hobert, 2009), contains multiple E-boxes (Fig. 6B). Interestingly, one of them is identical to the putative NGN-1:HLH-2 and HLH-16:HLH-2 binding site observed in the *ttx-3* initiator element (CATGTG, which is CACATG reverse complemented) and is conserved between different *Caenorhabditis* species. Mutating this binding site using CRISPR led to a reduction of CEH-10 expression level during the initiation phase (Fig. 6C). This suggests that NGN-1 and HLH-16 could directly regulate the initiation of *ceh-10* expression. There are also four putative HLH-3:HLH-2 binding sites identical to the one observed in the *ttx-3* initiator element. However, these sites are not conserved.

### NGN-1 and HLH-3 have an antagonistic effect on the expression of the proapoptotic gene *egl-1*

**Fig. 7.**
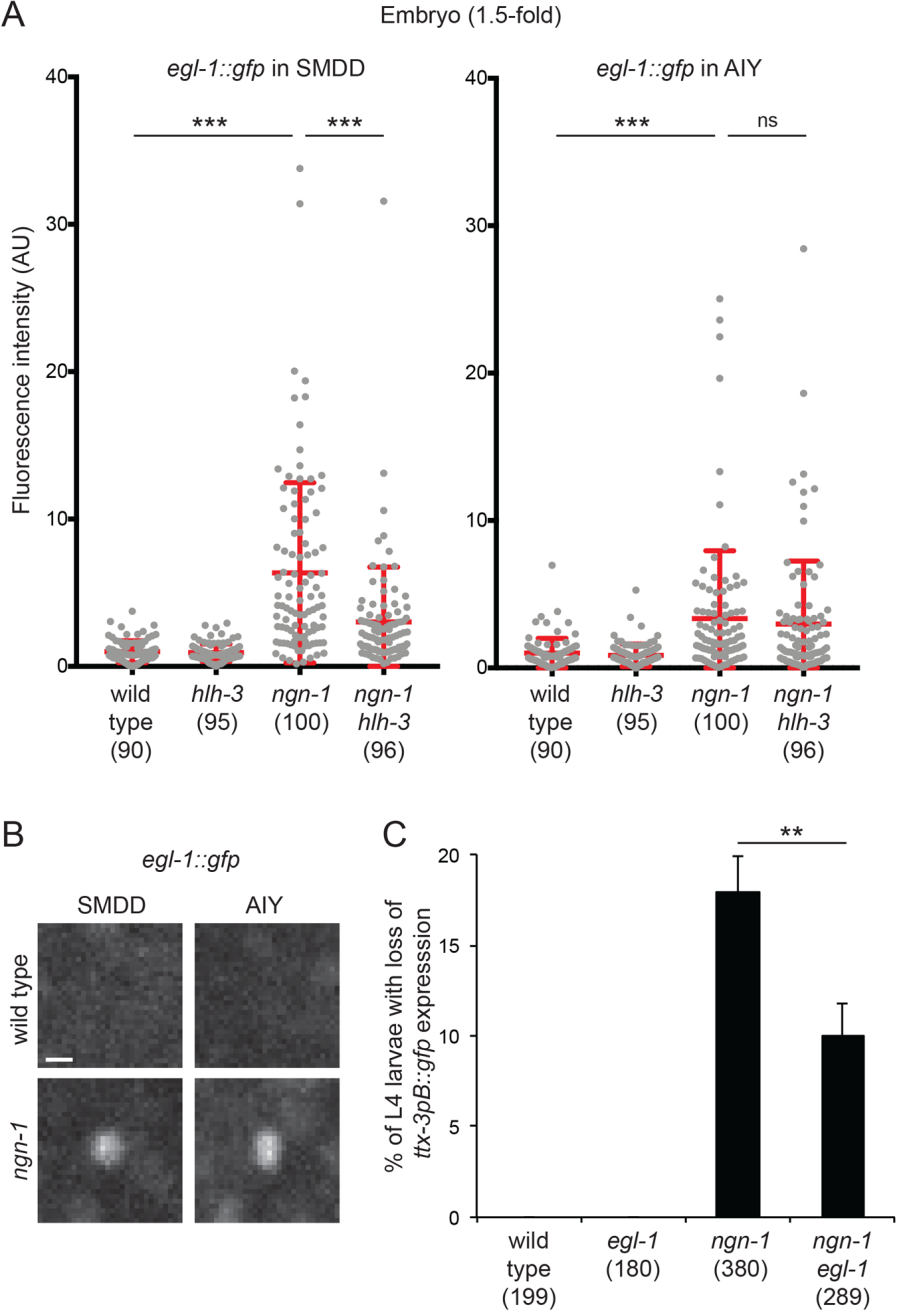
**Interaction between *ngn-1* and *egl-1*.** (A) Quantification of *egl-1::gfp (bcIs37)* expression in SMDD and AIY neurons at 1.5-fold embryonic stage in wild type or combinations of *ngn-1(ok2200)* and *hlh-3(tm1688)* mutants. Each grey dot represents one neuron. The number of neurons analyzed is presented below the genotype. Red bars represent mean and s.d.; Mann–Whitney test with Bonferroni correction for multiple comparisons (ns, not significant; ***P<0.001). (B) Pictures of *egl-1::gfp (bcIs37)* expression in SMDD and AIY neurons at 1.5-fold embryonic stage in wild type or *ngn-1(ok2200)* mutants; scale bar: 2 µm. (C) Percentage of L4 larvae with loss of *ttx-3pB::gfp (otIs173)* expression in at least one AIY neuron, in wild type or combinations of *ngn-1(ok2200)* and *egl-1(n1084n3082)* mutants. Error bars show standard error of proportion; numbers below the genotypes show numbers of animals analyzed; **P<0.01, Fisher's exact test.

In the *C. elegans* embryo, it was previously observed that HLH-3 is an activator of the expression of the proapoptotic gene *egl-1* that codes for a BH3-only protein (Thellmann et al., 2003). The HLH-3:HLH-2 complex directly activates *egl-1* expression by binding to E-boxes in its *cis*-regulatory regions. We therefore analyzed the effect of HLH-3 and NGN-1 on *egl-1* expression in the AIY lineage. We quantified the level of *egl-1* expression in the SMDD and AIY neurons at 1.5-fold embryonic stage using an *egl-1::gfp* reporter line (a nuclear histone::GFP fusion expressed under the control of the *egl-1 cis*-regulatory regions, *bcIs37* (Thellmann et al., 2003). In wild-type animals, *egl-1* expression is barely detectable in SMDD and AIY (Fig. 7A,B). However, in *ngn-1* mutants, we observed a strong derepression of *egl-1* expression. Interestingly, the derepression seems higher in SMDD than AIY (Fig. 7A), which mirrors the stronger expression of NGN-1 in SMDD than AIY (Fig. 2). This shows that NGN-1 acts as a repressor of *egl-1* expression. Consistent with a role of HLH-3 as an activator of *egl-1* expression, we observed no derepression of *egl-1* in *hlh-3* mutants (Fig. 7A). In addition, *hlh-3* loss of function suppresses the *egl-1* derepression observed in *ngn-1* mutants, at least in SMDD. Taken together, these data suggest that NGN-1 and HLH-3 have an antagonistic effect on *egl-1* expression.

In *ngn-1* mutants, we did not observe any sign of cell death of the AIY neuron before loss of AIY markers. While the level of derepression of *egl-1* may not be high enough to induce apoptosis, *egl-1* derepression could still interfere with AIY cell fate specification. To test whether *egl-1* derepression contributes to the *ngn-1* phenotype, we combined the *ngn-1* loss-of-function mutation with an *egl-1* loss-of-function mutation. We observed that the *egl-1* mutation suppresses partially the loss of AIY fate of the *ngn-1* mutant when monitored at larval stage (Fig. 7C). This suggests that part of the *ngn-1* phenotype is due to the detrimental action of *egl-1* derepression on AIY fate specification. The remaining part could reflect the direct action of *ngn-1* on the initiation of *ceh-10* expression. Consistent with this hypothesis, we observed that the *egl-1* mutation does not suppress the effect of *ngn-1* mutants on *ceh-10* initiation (Fig. S5). We also noticed that the *egl-1* mutation does not suppress the effect of *ngn-1* mutants on AIY axonal projection or AIY position (Fig. S6).

To conclude, our study shows that the different neural bHLHs act together to ensure a robust activation of the terminal selector transcription factors, while, at the same time preventing the unwanted activation of a deleterious gene, *egl-1*.

## DISCUSSION

In this study, we analyzed how multiple neural bHLHs ensure the robustness of neuron-type specification. We showed that neural bHLHs act together to set the correct level of activation for terminal selector transcription factors. We also established that the use of multiple neural bHLHs prevents the unwanted activation of a deleterious gene, *egl-1*.

### The combined action of several neural bHLHs sets the correct level of terminal selector activation

**Fig. 8.**
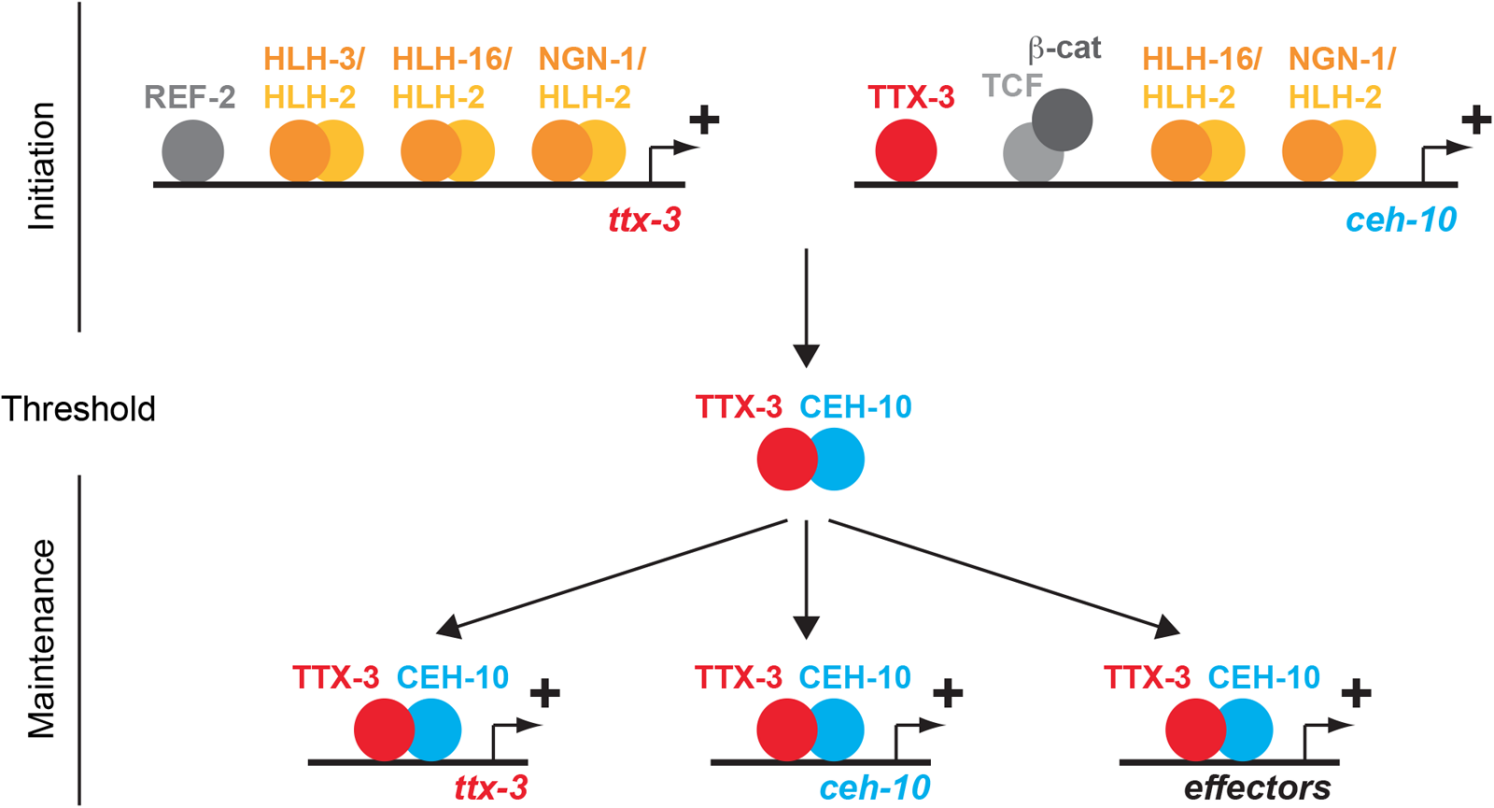
**Fine-tuning of terminal selector initiation by neural bHLH factors.** The neural bHLH factors regulate the level of expression of the terminal selectors *ttx-3* and *ceh-10* during the initiation phase. This allows the level of TTX-3 and CEH-10 proteins to reach a threshold above which TTX-3 and CEH-10 can maintain their expression, as well as trigger and maintain the terminal differentiation of the AIY neuron.

In this paper, we showed that the three neural bHLH factors HLH-3 (Achaete-scute), HLH-16 (Olig) and NGN-1 (Neurogenin) regulate the initial expression of the terminal selectors TTX-3 (Lhx2/9) and CEH-10 (VSX1/2) in the AIY neuron lineage (Fig. 8). HLH-3, HLH-16 and NGN-1 likely act as heterodimers with the E-protein HLH-2 via E-boxes present in the *cis*-regulatory regions of *ttx-3* and *ceh-10*. Following this initiation phase, the neural bHLH factors disappear from the AIY neuron. TTX-3 and CEH-10 form a complex and self-maintain their expression via binding sites present in their *cis*-regulatory regions, thereby locking in the AIY fate (Bertrand and Hobert, 2009; Wenick and Hobert, 2004). This type of positive feedback loop generates bistable ON-OFF states depending on whether a minimal threshold is reached or not (Chalancon et al., 2012). Interestingly, we observed that the three neural bHLHs are required to ensure the proper level of expression for *ttx-3* and *ceh-10* during the initiation phase. In the absence of one neural bHLH, the expression level of *ttx-3* or *ceh-10* may not be high enough in some AIY neurons to trigger the self-maintenance loop, leading to partially penetrant misspecification of the AIY neuron. Therefore, having multiple neural bHLHs secures a 100% efficient neuronal specification by fine-tuning the level of terminal selector initiation. In addition to the regulation of *ttx-3* and *ceh-10* expression, neural bHLHs could also influence the expression of each other via cross-regulation as observed in other systems (Baker and Brown, 2018).

What does AIY become in bHLH mutants after losing *ttx-3* and *ceh-10* expression? In *ttx-3* and *ceh-10* mutants, while AIY loses its type-specific identity, it remains a neuron expressing pan-neuronal markers (Altun-Gultekin et al., 2001). We have previously observed that in *hlh-3* mutants, when *ttx-3* expression is lost, a cell is still present in L4 larvae at the position of AIY and still expresses a pan-neuronal marker (Murgan et al., 2015). However, in *ngn-1* and *hlh-16* mutants, neurons are mispositioned at L4 larval stage making this analysis impossible. Therefore, the destiny of AIY after losing *ttx-3* and *ceh-10* expression in *ngn-1* and *hlh-16* mutants remains an open question.

### Use of several neural bHLHs prevents the spurious activation of deleterious genes

A simpler strategy to ensure a robust neuron-type specification could be to use higher quantities of a single neural bHLH factor instead of using multiple neural bHLHs at lower levels. However, our data suggest that such a strategy may be deleterious. HLH-3 has been previously shown to be an activator of *egl-1* expression, a proapoptotic gene of the BH3-only family (Thellmann et al., 2003). Here we observed that NGN-1 acts as a repressor of *egl-1* expression, antagonizing HLH-3 action, and that *egl-1* is detrimental to the specification of the AIY neuron. These data allow us to propose a hypothesis for why multiple neural bHLH factors are used for a single neuronal specification event. Use of a single neural bHLH (like HLH-3) at reasonable level may not provide sufficient neural bHLH activity to secure a 100% efficient neuronal specification. Increasing the level of this neural bHLH may lead to the ectopic activation of deleterious genes, such as *egl-1*. On the contrary, adding a second neural bHLH (like NGN-1) allows a 100% efficient neuronal specification, while avoiding deleterious gene activation.

Why do HLH-3 and NGN-1 have opposite effects on *egl-1*? This may be due to the fact that HLH-3 and NGN-1 bind slightly different types of E-boxes. While HLH-3 binds *egl-1 cis*-regulatory regions (Thellmann et al., 2003), NGN-1 may not bind to them. Instead, NGN-1 may bind to the *cis*-regulatory regions and activates the expression of another gene that acts as a repressor of *egl-1*. Another possibility is that NGN-1 binds *egl-1 cis*-regulatory regions and that the presence of a specific cofactor on *egl-1 cis*-regulatory regions turns NGN-1 into a repressor. Interestingly, a recent RNA sequencing study showed that the majority of differentially expressed genes in *ngn-1* mutants are upregulated supporting the idea that NGN-1 may indeed be able to act as a repressor (Christensen et al., 2020).

### Robustness of neuronal cell fate specification

In several systems, it has been observed that redundancies between transcription factors or *cis*-regulatory regions play an important role in ensuring the robustness of gene expression during development (Lagha et al., 2012; Raj et al., 2010). The expression of terminal selectors has to be tightly controlled, because of their ability to self-activate and to regulate large batteries of neuron-type specific effector genes. Interestingly, it has been previously shown that the combined action of different homeodomain transcription factors is required for the efficient activation of the terminal selector MEC-3 in *C. elegans* touch receptor neurons (Zheng et al., 2015). Here, we observed that the collaboration of multiple neural bHLH factors ensures the correct level of initiation of the terminal selectors TTX-3 and CEH-10 in a *C. elegans* cholinergic interneuron. This suggests that fine-tuning of the expression level via the combined action of multiple upstream transcription factors may be a general principle in the initiation of terminal selector expression, allowing a robust neuronal specification.

Neural bHLH factors play a key role in the early steps of neuron-type specification in many animals (Baker and Brown, 2018; Bertrand et al., 2002; Hartenstein and Stollewerk, 2015). In vertebrates, it has been observed that different neural bHLH factors can be coexpressed in the same neuronal progenitor or postmitotic neuron (Baker and Brown, 2018). The combined action of several neural bHLHs, similar to the one described here in *C. elegans*, may therefore also play a role in the robustness of neuronal specification in animals with more complex nervous systems like vertebrates.

## MATERIALS AND METHODS

### *Caenorhabditis elegans* strains

All experiments were performed on *C. elegans* hermaphrodites. Genotyping primers for *ngn-1*, *hlh-3*, *hlh-16*, *hlh-2*, *egl-1*, *rrf-3* and *ttx-3* mutants are presented in Table S2. The *ttx-3endo::yfp (vba3)* and *ceh-10endo::yfp (vba1)* strains were generated by inserting the *yfp* coding region in frame with a SGGGGS linker at the C-term end of the endogenous *ttx-3* and *ceh-10* genes using CRISPR genome engineering with *unc-22* or *rol-6* co-CRISPR strategies (Arribere et al., 2014; Kim et al., 2014). The sequence targeted by the guide RNA was: CAGAGGTGGTGTGTTGAGCTGG (PAM underlined) for *ttx-3* and CGGAAAAATAGAGTTACATGGG for *ceh-10*. In the *ceh-10endo::yfp (vba1)* strain, the CATGTG E-box was subsequently mutated to AATTCG (creating an EcoRI site) by CRISPR generating the *vba28* allele. The sequence targeted by the guide RNA was: TACAAAGTCAGCTGAGGCGGCGG.

### RNAi screen

The effect of RNAi clones targeting bHLH factors was tested on AIY neurons labeled with *ttx-3pB::gfp (mgIs18)* in two different genetic backgrounds: *rrf-3(pk1426)* or *rrf-3(pk1426); hlh-2(bx115)*. *rrf-3(pk1426)* increases the general efficiency of RNAi treatments (Simmer et al., 2003), and *hlh-2(bx115)* is a hypomorphic mutation (Portman and Emmons, 2000) that does not induce AIY defects on its own but increases the effect of knockdowns of its heterodimerizing bHLH partners. 37 bHLH factors were tested (out of the 42 bHLH factors present in the *C. elegans* genome; Grove et al., 2009) (Table S1). The source of RNAi clones is indicated in Table S1: Ahringer library, Vidal library, *hlh-2* (Karp and Greenwald, 2003) and *hlh-14* (Poole et al., 2011). Hermaphrodites at the L4 stage were grown on bacteria expressing the RNAi clone of interest and the effect on AIY neurons was then analyzed in their progeny.

### *hlh-2* RNAi treatments

L4 stage hermaphrodites were grown on bacteria harboring a plasmid to express dsRNA (L4440 as negative control, pKM1196 for *hlh-2*; Fraser et al., 2000; Karp and Greenwald, 2003). Their F1 offspring was then analyzed. The genotypes of the strains used for RNAi were: *ceh-10(vba1); rrf-3(pk1426)* for *ceh-10* expression [the *rrf-3(pk1426)* mutation increases RNAi efficiency; Simmer et al., 2003], and *ttx-3(vba3); ngn-1(ok2200); hlh-16(ot711); hlh-3(tm1688)* for *ttx-3* expression.

### Microscopy

Standard analyses were performed using a Zeiss Axioplan II epifluorescence microscope equipped with a Zeiss AxioCam MRm camera, or a Nikon Eclipse Ti2E epifluorescence microscope equipped with a Hamamatsu ORCA-Flash4.0 camera.

Quantification of fluorescence levels in *ttx-3endo::yfp (vba3)*, *ceh-10endo::yfp (vba1)* or *egl-1::gfp (bcIs37)* strains were performed using a Nikon Eclipse Ti microscope equipped with a Yokogawa CSU-X1 spinning disc module, 480 nm/515 nm/561 nm lasers, and a Photometrics Evolve EMCCD camera. Embryos were mounted on a 5% agar pad between a slide and a coverslip. 3D image stacks were acquired and the fluorescence intensity inside the nucleus of interest was then measured using Fiji. This value was then corrected for background autofluorescence.

### Statistics

Statistical analysis was performed using GraphPad Prism. For comparisons of fluorescence levels, a non-parametric two-tailed Mann–Whitney *U*-test was performed. For comparisons of proportions, a two-tailed Fisher's exact test was performed. A Bonferroni correction was applied when multiple comparisons were performed.

## Supplementary Material

Supplementary information
