# Supplementary material for: Multiple neural bHLHs ensure the precision of a neuronal specification event in Caenorhabditis elegans
Source: Biol Open. 2021 Dec 23;10(12):bio058976. doi: 10.1242/bio.058976 (PMC8713986; doi:10.1242/bio.058976)
Supplement: Supplementary information [file biolopen-10-058976-s1.pdf]

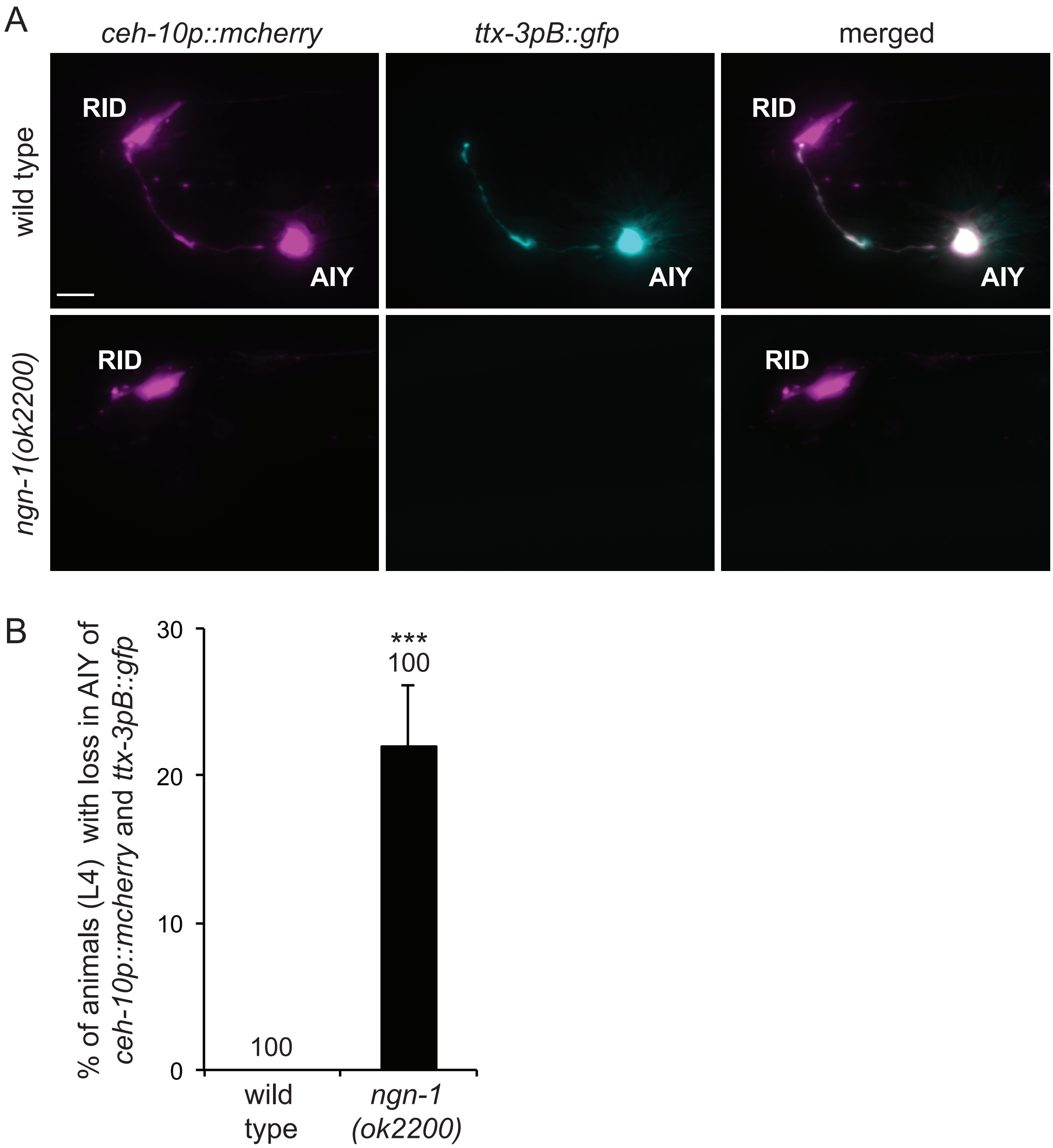

Fig. S1. Effect of *ngn-1* on *ceh-10* and *ttx-3* co-expression in AIY.
(A) Co-expression of *ceh-10p::mcherry (hpIs292)* and *ttx-3pB::gfp (mgIs18)* in the AIY neuron of wild type or *ngn-1* mutant larvae (L4). *ngn-1* affects *ceh-10p::mcherry* and *ttx-3pB::gfp* expression in the AIY neuron, but not *ceh-10p::mcherry* expression in another neuron, RID. Lateral view, anterior is left, dorsal is up, scale bar = 10 μm.
(B) Percentage of animals with a loss of *ceh-10p::mcherry (hpIs292)* and *ttx-3pB::gfp (mgIs18)* expression in at least one AIY in wild type or *ngn-1* mutants, in late larvae (L4). In one AIY, when *ceh-10p::mcherry* expression is absent, *ttx-3pB::gfp* expression is also absent, and vice versa. Error bars show standard error of proportion; numbers above the bars show numbers of animals analyzed; *** $p<0.001$, Fisher's exact test.

Biology Open • Supplementary information

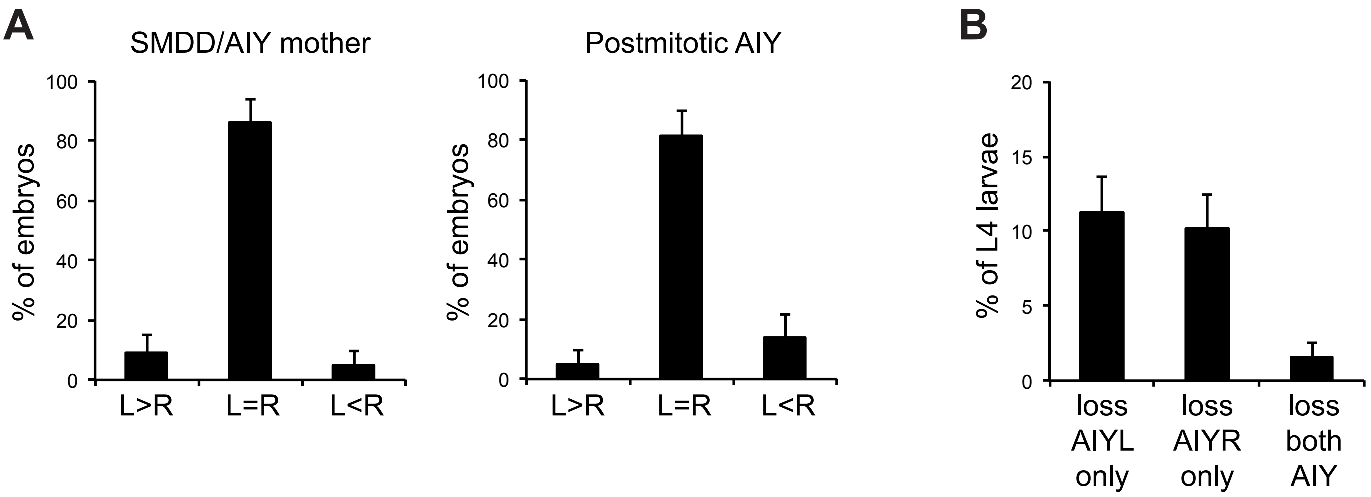

Fig. S2. NGN-1 expression and loss of *ttx-3* expression in *ngn-1* mutants do not display left-right asymmetries.

(A) Left-right asymmetry in the expression of the NGN-1 protein tagged with GFP (*nIs394*) in embryos at epidermal enclosure stage before or after the terminal division of the SMDD/AIY mother cell. L>R: expression higher in the left lineage, L=R: expression identical between left and right lineages, L<R: expression lower in the left lineage. The AIY lineage is identified with *hlh-16p::mcherry (stIs10546)*. Error bars show standard error of proportion; n = 21 embryos analyzed for SMDD/AIY mother and 21 embryos analyzed for postmitotic AIY.
(B) Percentage of *ngn-1(ok2200)* larvae (L4 stage) with a loss of *ttx-3pB::gfp (otIs173)* expression in the left AIY only, right AIY only, or both AIY. Error bars show standard error of proportion, n = 186 larvae analyzed.

Biology Open • Supplementary information

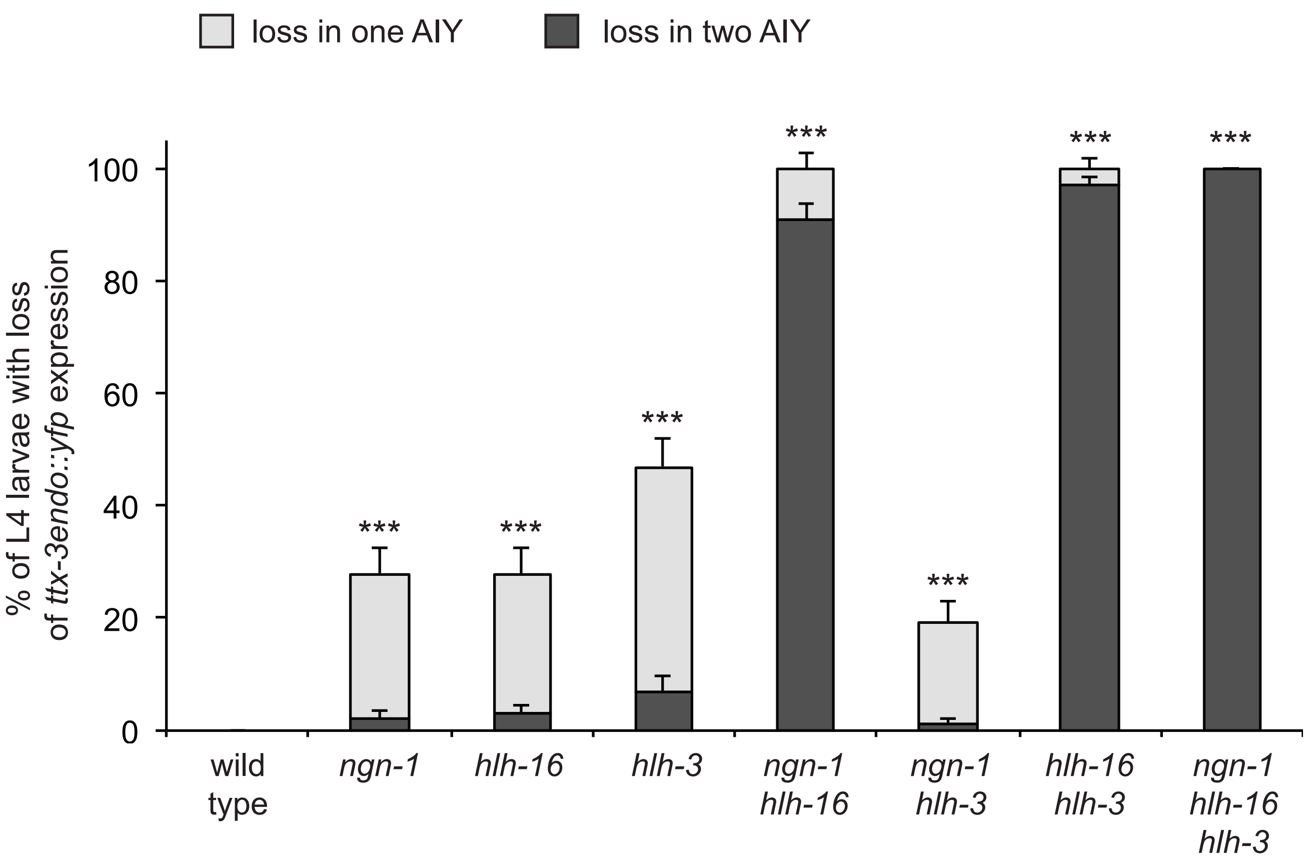

Fig. S3. Effect of combinations of bHLH mutants on *ttx-3endo::yfp* expression at larval stage. Percentage of animals with loss of *ttx-3endo::yfp (vba3)* expression in one or both AIY neurons at late larval stage (L4) in wild type or combinations of bHLH mutants. Error bars show standard error of proportion; n=100 neurons analyzed for each genotype; Fisher's exact test with Bonferroni correction for multiple comparisons (*** $p<0.001$).

Biology Open • Supplementary information

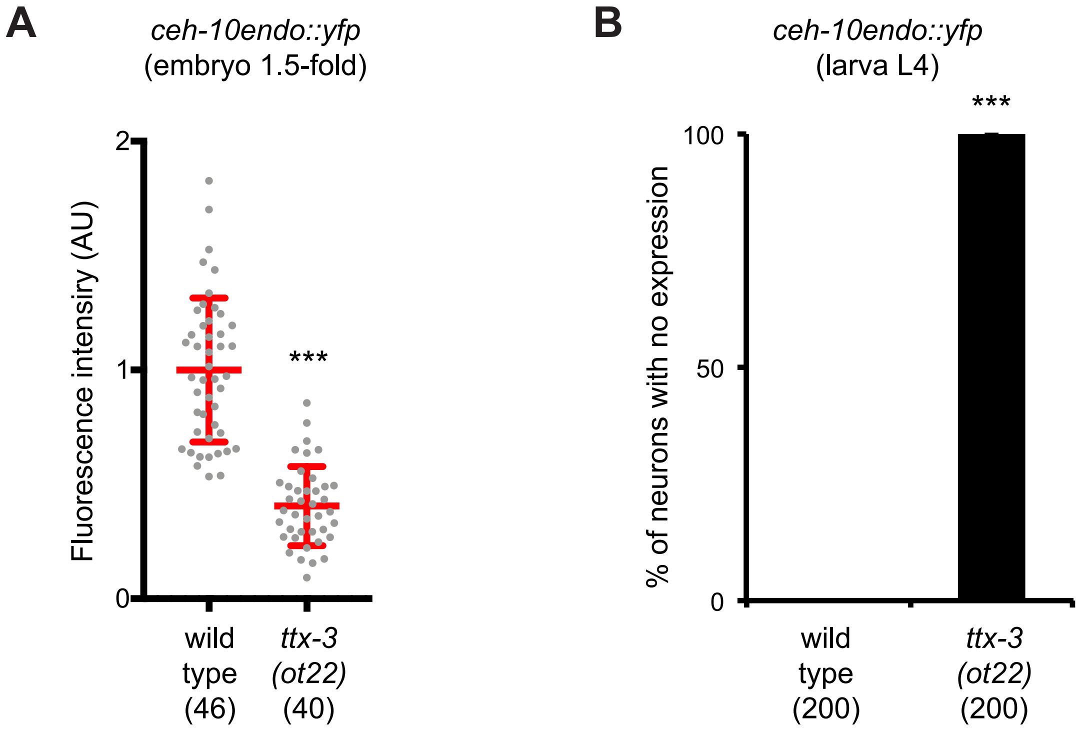

Fig. S4. Effect of *ttx-3* mutants on *ceh-10endo::yfp* expression.
(A) Quantification of the fluorescence levels of *ceh-10endo::yfp (vba1)* in AIY neurons at 1.5-fold embryonic stage in wild type or *ttx-3(ot22)* mutants. Each grey dot represents one neuron. The number of neurons analyzed is presented below the genotype. Red bars = mean and SD, Mann-Whitney test (*** $p<0.001$).
(B) Percentage of AIY neurons with no expression of *ceh-10endo::yfp (vba1)* at L4 larval stage in wild type or *ttx-3(ot22)* mutants. The number of neurons analyzed is presented below the genotype; error bars show standard error of proportion; Fisher's exact test (*** $p<0.001$).

Biology Open • Supplementary information

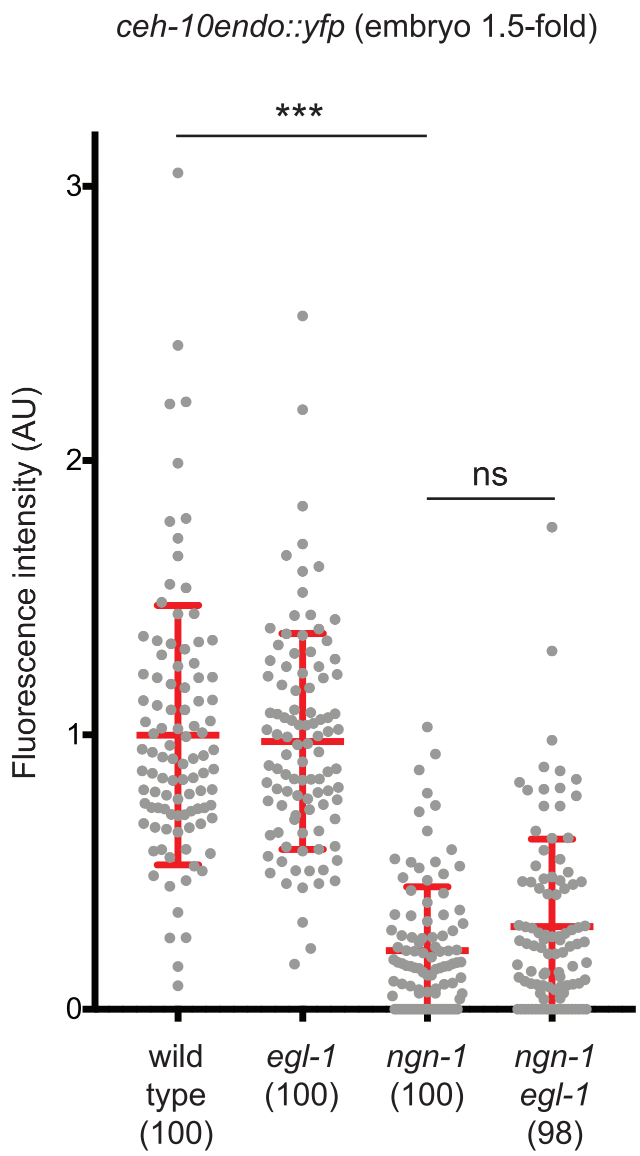

Fig. S5. Effect of *ngn-1* and *egl-1* on *ceh-10* initiation level.
Quantification of the fluorescence levels of *ceh-10endo::yfp (vba1)* in AIY neurons at 1.5-fold embryonic stage in wild type or combinations of *ngn-1(ok2200)* and *egl-1(n1084n3082)* mutants. Each grey dot represents one neuron. The number of neurons analyzed is presented below the genotype. Red bars = mean and SD, Mann-Whitney test with Bonferroni correction for multiple comparisons (ns: not significant, *** p<0.001).

Biology Open • Supplementary information

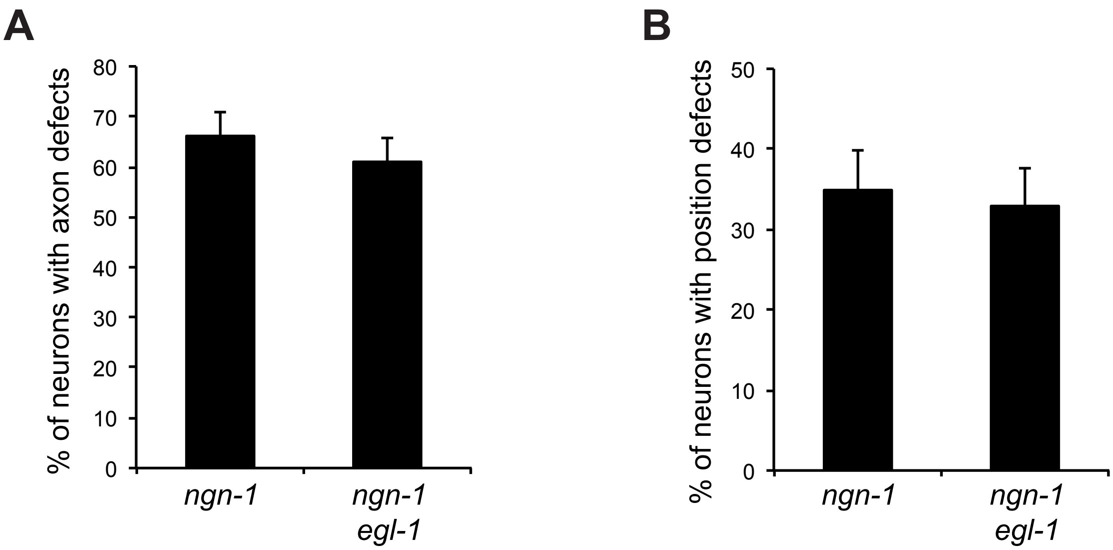

Fig. S6. *egl-1* does not suppress axon and position defects of *ngn-1* mutants.
(A) Percentage of AIY neurons expressing *ttx-3pB::gfp (otIs173)* with axonal projection defects in *ngn-1(ok2200)* single mutants or *ngn-1(ok2200); egl-1(n1084n3082)* double mutants at late larval stage (L4). Axonal projection is considered defective if the axon stops before reaching the middle of the nerve ring, n = 100 neurons analyzed for each genotype.
(B) Percentage of AIY neurons expressing *ttx-3pB::gfp (otIs173)* with cell body position defects in *ngn-1(ok2200)* single mutants or *ngn-1(ok2200); egl-1(n1084n3082)* double mutants at late larval stage (L4). Position is considered defective if the cell body is located anterior to the posterior bulb of the pharynx, n = 100 neurons analyzed for each genotype.

Table S1. RNAi screen on bHLH factors.
Presence (+) or absence (-) of defects of AIY labeled with *ttx-3pB::gfp* (*mgIs18*). bHLH clones were tested in two different genetic backgrounds: *rrf-3(pk1426)* and *rrf-3(pk1426); hlh-2(bx115).*

ORF	Gene name	Clone source	Clone name	Defects in *rrf-3(pk1426)*	Defects in *rrf-3(pk1426); hlh-2(bx115)*
C25A1.11	*aha-1*	Vidal Library	mv_AAB99999	-	-
C41G7.5	*ahr-1*	Ahringer Library	I-4P19	-	-
C15C8.2	*cky-1*	Vidal Library	mv_C15C8.2	-	-
C34E10.7	*cnd-1*	Ahringer Library	III-2B18	-	-
F38A6.3	*hif-1*	Ahringer Library	V-13O05	-	-
B0304.1	*hlh-1*	Ahringer Library	II-3J04	-	-
ZK682.4	*hlh-10*	Ahringer Library	V-6F01	-	-
F58A4.7	*hlh-11*	Ahringer Library	III-5K17	-	-
C28C12.8	*hlh-12*	Ahringer Library	IV-4G20	-	-
F48D6.3	*hlh-13*	Ahringer Library	X-2H09	-	-
C18A3.8	*hlh-14*	Poole et al.		-	-
C43H6.8	*hlh-15*	Ahringer Library	X-1J06	-	-
DY3.3	*hlh-16*	Ahringer Library	I-4I02	+	+
F57C12.3	*hlh-19*	Ahringer Library	X-1E19	-	-
M05B5.5	*hlh-2*	Karp and Greenwald	pKM1196	+	nd
C17C3.8	*hlh-26*	Vidal Library	mv_C17C3.8	-	-
F31A3.2	*hlh-28*	Ahringer Library	X-7H22	-	-
F31A3.4	*hlh-29*	Ahringer Library	X-7J02	-	-
T24B8.6	*hlh-3*	Ahringer Library	II-6F03	-	+
W02C12.3	*hlh-30*	Ahringer Library	IV-2C11	-	-
F38C2.8	*hlh-31*	Ahringer Library	IV-7J16	-	-
Y39A3CR.6	*hlh-33*	Ahringer Library	III-7I11	-	-
T01D3.2	*hlh-34*	Ahringer Library	V-9C07	-	-
T05G5.2	*hlh-4*	Vidal Library	mv_T05G5.2	-	-
T15H9.3	*hlh-6*	Ahringer Library	II-6J18	-	-
C02B8.4	*hlh-8*	Ahringer Library	X-4M03	-	-
C44C10.8	*hnd-1*	Ahringer Library	X-5J01	-	-
T14F9.5	*lin-32*	Ahringer Library	X-1D20	-	-
R03E9.1	*mdl-1*	Vidal Library	mv_R03E9.1	-	-
T20B12.6	*mml-1*	Vidal Library	mv_T20B12.6	-	-
T19B10.11	*mxl-1*	Ahringer Library	V-7N05	-	-
F40G9.11	*mxl-2*	Ahringer Library	III-1G23	-	-
F46G10.6	*mxl-3*	Ahringer Library	X-6C16	-	-
Y69A2AR.29	*ngn-1*	Ahringer Library	IV-8L18	+	+
T01E8.2	*ref-1*	Ahringer Library	II-7O13	-	-
Y47D3B.7	*sbp-1*	Ahringer Library	III-6C01	-	-
Y16B4A.1	*unc-3*	Ahringer Library	X-6J06	-	-

Biology Open • Supplementary information

Table S2. Genotyping primers.
For large deletions, external (Ex) and internal (In) primers were used in forward (F) or reverse (R) orientation for PCR with the combinations: ExF/ExR, ExF/InR, InF/ExR. For point mutations or small deletions a pair of forward (F) and reverse (R) primers was used for PCR amplification before sequencing.

Allele	Primers
ngn-1(tm2446)	ExF: GCCAAGTGAGAGCAAATTTGAC<br>ExR: CCAGCTCTTCCAGATGAGCC<br>InF: CGTCTCGCAGCACTGAGAGG<br>InR: CCTCTCAGTGCTGCGAGACG
ngn-1(ok2200)	ExF: ATTTCCGACGACGAACTGTC<br>ExR: ATCTCCAGACTGGTGTTCCG<br>InF: ACAGCTGTACAATACCATGAG<br>InR: GTTCGGATTTCTAAGCCACC
hlh-3(tm1688)	ExF: CACACAAAACAAGAACCGGCCAC<br>ExR: GACATTCCGAGTTGCTTCTGCAG<br>InF: ATTCAGAAGCTCCAGCGCAATGC<br>InR: ATGTGATTGCAATTCTGTCACCATG
rrf-3(pk1426)	ExF: TGCGATTGCGATTGGAAACTGCC<br>ExR: CTCTTGATACTTGCAGCATGTCC<br>InF: AAGAATGAGTCACGCTCATTTGGC<br>InR: CGTTTCCATATTGAGAACCTTCGAC
hlh-2(bx115)	F: GGAATGCCACCAGGATCAAGTGC<br>R: GGTTTCCTTTTCATTCCAGCCATCAC
hlh-16(ot711)	F: GCCGACAGCTTCATCTCCG<br>R: TGAAGCCCTTCCACCGAAG
egl-1(n1084n3082)	F: GATCTTCTACCAATGTCCAACG<br>R: ATGGAAGAGGCTTCTGTCGG
ttx-3(ot22)	F: CTTAACCCGCTTTGTACCGAGTAG<br>R: GTACTGAACCAGTATTTTCATGCGTG
